# DPAS: disease-associated peptide anomaly score for identifying pathogenic peptides via one-class learning

**DOI:** 10.1038/s41598-026-40099-0

**Published:** 2026-02-15

**Authors:** Zoya Khalid, Razia Khalid, Osman Ugur Sezerman

**Affiliations:** 1 Department of Biosciences, COMSATS University, Islamabad, Pakistan; 2 National University of Computer and Emerging Sciences, NUCES, FAST, Islamabad, Pakistan; 3 Faculty of Medicine, Department of Biostatistics and Medical Informatics, Acibadem University, Istanbul, Turkey

**Keywords:** Biomarkers, Computational biology and bioinformatics

## Abstract

Predicting disease-associated peptides is a challenging task in bioinformatics, mostly hindered by the lack of reliable negative datasets, leading to biased predictions. In this study, we propose a one-class classification approach that focuses exclusively on positive-labeled data. We employed three classifiers namely One-Class Support Vector Machines (OCSVM), Isolation Forest, and Autoencoders to classify disease-associated peptides, with Autoencoders yielding the best results. The Autoencoders trained on the positive dataset effectively differentiated the inliers from outliers which is further evaluated by mean reconstruction errors. Our method combines various sequence based features together. This framework provides an efficient solution for predicting disease-associated peptides that also overcomes the traditional binary classification approaches. To enhance interpretability and peptide prioritization, we introduce a new scoring metric Disease Peptide Anomaly Score (DPAS) which combines model-derived anomaly scores with feature importance values obtained using SHAP (SHapley Additive exPlanations). DPAS facilitates the ranking of peptides based on their likelihood of being disease-associated, offering a robust and interpretable approach for peptide biomarker discovery.

**Supplementary Information:**

The online version contains supplementary material available at 10.1038/s41598-026-40099-0.

## Introduction

Peptides are a short chain amino acids that are cleaved from large proteins and are increasingly getting popular especially among pharmaceuticals. Nowadays, peptide based drugs are a new trend in therapeutics that have gained the attention of researchers. Peptide-based biomarkers play a crucial role in disease diagnosis and prognosis^1^. Conversely, bringing a new peptide in market poses a challenge both financially and experimentally as it’s a costly trade also, the poor pharmacokinetic properties further increases the complexity. Here, the computational approaches comes as a savior which tries to complement the experimental approaches by accelerating the process of accurate predictions and also aids in picking the suitable candidates for subsequent experimental validations. Many computational approaches uses sequence based function prediction of the peptides by mapping various biological properties to it and identifying the disease associated peptides.

Peptides are categorized as natural or synthetic out of which natural peptides are studied considerably as compared to synthetic ones. Both these peptides have proteinogenic means natural amino acids and non-proteinogenic means modified amino acids as building blocks which can boost the pharmacokinetic properties for better therapeutics^2^. As one of the noticeable properties of peptides is the high level of specificity despite the challenges associated with effectively targeting them to specific sites, makes it a promising candidate for therapeutics^3^.

To develop classification models supervised learning methods are very successful among computational researchers. But in biological data analysis the negative dataset becomes a limitation. For instance, the classification model to identify protein binding sites, proteins that binds to a potential target are labelled as positive example, while proteins that do not bind are labelled as negative examples. Conversely, the lack of binding could be be due to unsatisfied binding conditions, which does not certainly mean that these proteins are a part of negative examples. The challenge of selecting appropriate negative examples has been featured by various researchers. Li et al., reported that the studies on protein-protein interactions often struggles in identifying the non-interacting pairs^4^. As advances in this field, the non-interacting pairs are mostly incorrectly classified with interacting pairs always outnumbers with less information, making the dataset imbalanced which pose further challenges to tackle. Same challenges could be found in the similar research areas like drug drug interactions, lncRNAs, gene function and many more^5^.

Recently, many studies have reported on identifying peptide-disease association using machine learning models. One reported study^6^, utilized the multi feature fusion technique to predict anti-inflammatory peptides (AIPCs). The method opted for voting classifier to predict the AIPCs, the method showed great accuracy in predictions. Next, few other studies reported on anticancer peptide prediction using machine learning that extracted features from the protein sequence and reported the accuracy of 91% by applying support vector machines^7^. Following, another paper utilized support vector machines on the pseudo-amino acid composition and local alignment as features and obtained the accuracy of 89%^8^. Another study reported by Xu et al.^8^utilized 400 dimensional feature set and obtained an accuracy of 91.86%^9^. Despite their high reported performances, the reported studies rely heavily on supervised learning methods that require well-defined positive and negative datasets which are often unavailable or manually constructed in biological domain. This can lead to biased models with limited generalizability to real-world, imbalanced data. Moreover, these models may overfit known patterns and fail to detect rare or novel peptides. In contrast, one-class classification addresses these limitations by learning solely from positive examples, offering a more realistic and robust approach for peptide-disease association prediction.

In this study, we postulate that disease-associated peptides show a coherent and structured feature distribution. By modeling this distribution using one-class learning approaches, we aim to identify deviations from it characterizing peptides whose attributes differ significantly from the positive class and may therefore be treated as potential outliers. To obtain the dataset we queried the Mutated Peptide- Database. Various sequence based features were extracted from the mutated peptide sequences and the feature matrix is generated. The current study has used one-class classification method that enhances the prediction performance over binary class classification. This also reduces the problem of generating noisy samples as negative dataset. This method works well for identifying the disease associated peptides as obtaining the negative dataset is always a challenge which sometimes leads to biased predictions. The hybrid feature set was generated that combines 6 distinct features together. Three classifiers namely onclassSVM, Isolation Forest and autoencoders were trained.

## Materials and methods

### Data collection

First, we downloaded the dataset from XMan a Homo sapiens Mutated-Peptide Database^10^. We then only selected the missense mutation type data. We label this data as positive data. We got total 767,305 examples as positive dataset. Due to the large size of the dataset (~ 1.5 million examples), we divided it into 10 chunks to perform feature extraction without exceeding computational resources. Features were extracted chunk-wise and then concatenated to form the complete dataset. Principal component analysis (PCA) was subsequently applied to the combined feature set, ensuring a consistent feature space across all samples for model training.

**Fig. 1:**
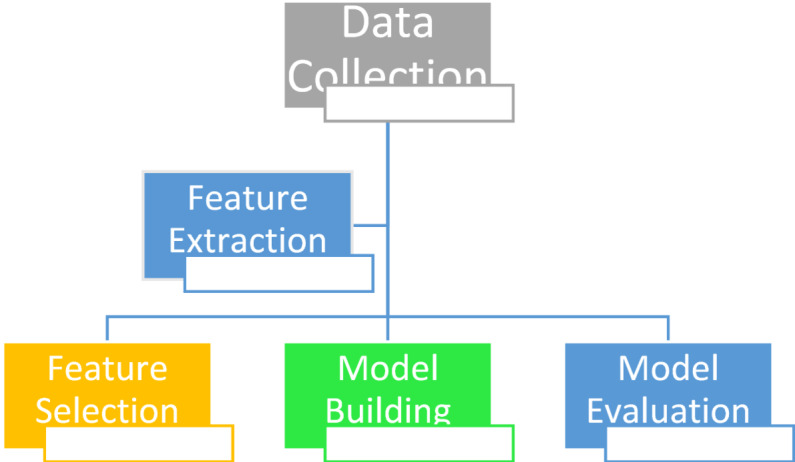
A complete flowchart of the methodology.

The methodology workflow is provided in Fig. 1.

### Feature extraction

The following sequence based features were extracted from the peptide sequences.

### Amino acid composition

AAC is defined as the fraction of each amino acid in the given peptide sequence, and it was calculated using the following formula.

$$\:\mathrm{A}\mathrm{A}\mathrm{C}\left(i\right)=\frac{\mathrm{F}\mathrm{r}\mathrm{e}\mathrm{q}\mathrm{u}\mathrm{e}\mathrm{n}\mathrm{c}\mathrm{y}\:\mathrm{o}\mathrm{f}\:\mathrm{a}\mathrm{m}\mathrm{i}\mathrm{n}\mathrm{o}\:\mathrm{a}\mathrm{c}\mathrm{i}\mathrm{d}\left(i\right)}{\mathrm{L}\mathrm{e}\mathrm{n}\mathrm{g}\mathrm{t}\mathrm{h}\:\mathrm{o}\mathrm{f}\:\mathrm{t}\mathrm{h}\mathrm{e}\:\mathrm{p}\mathrm{e}\mathrm{p}\mathrm{t}\mathrm{i}\mathrm{d}\mathrm{e}}$$

where *i* represents any of the 20 natural amino acids. AAC has a fixed length of 20 corresponding to the 20 amino acids each position represents the frequency^11^. Each position in a sequence window is represented as 20 dimensional vector that is filled with 1’s and 0’s indicating the presence or absence of a particular amino acid in the provided sequence. The amino acids are also grouped by using Sezerman grouping^12^that creates an 11 dimensional vector for each position of amino acid. This grouping method is beneficial for the rare amino acids which when comes in a group may become frequent.

### Physicochemical properties

#### Volume measure

We used amino acid volumes from Kharakoz’s estimates^13^to find out volume of the peptide sequence. For this, we determined the Volume measure value for each amino acid in a sequence. Then these values are summed up and later divided by the length of the peptide sequence to get a single feature.

#### Hydrophobicity measure

To estimate the average hydrophobicity of the peptide sequence we used the hydrophobicity scales from Hopp & Woods^14^. For each amino acid in a sequence we first calculated the Hydrophobicity measure value. Then we have summed all these values and divide them by the length of sequence to get a single feature representing the average hydrophobicity of the entire peptide sequence.

Further, we used Pfeature tool^15^to calculate the physico-chemical properties composition from the peptide sequence. We used 5 physico-chemical properties named: (1) hydrophobic (2) hydrophilic (3) neutral (4) positively charged (5) negative-charged from the tool which added 5 features to the feature vector.

### Shannon entropy

The Pfeature webserver was utilized to compute shanon entropy where we used SER function to calculate the Shannon entropy of the peptide sequence. This feature computes the variability in the peptide sequence and its contribution in disease association. This function computed the Shannon entropy at residue level.

### Repetitive residue information (RRI) (20 vector) (using p-feature tool)

We used Pfeature tool to calculate the Repetitive Residue Information of the peptide sequence. It counts the occurrence of amino acids in a peptide sequence that occurs multiple times which may provide functional significance.

### Dipeptide composition

DPC is defined as the frequency of dipeptides normalized against 400 possible dipeptide combinations of the 20 amino acids. We utilized the p-feature tool to calculate this feature. The DPC feature vector has a fixed length of 400, with each component representing the normalized count of a specific dipeptide. This feature captures local sequence patterns and interactions, providing insights into the relationships between consecutive amino acids in the sequence.

### Extracting motifs

Motifs are the short sequence pattern that are conserved and caries structural functional significance. We used MEME^16^web server to find the motifs in peptide sequences. We found total 158 motifs in the peptide sequences. We then used the MAST^17^Web server to search positive and negative sequences for matches to a set of motifs. Each sequence is represented by a 158-dimentional feature vector. If the motifs found in positive peptide sequence have higher E-value than the threshold it will be marked as 1 in positive sequence and 0 otherwise. In case of missing motifs all corresponding motifs vector were left 0.

### Feature importance and selection

For this study we have extracted a diverse set of features including physicochemical properties, Shannon entropy, volume measures, motifs, amino acid composition, and dipeptide composition. As we have extracted these features on a mutated dataset all these sequence based features are important to reflect the sequence variations and its influence in identifying disease associated peptides. Among them, motifs plays a critical role in the prediction as they capture the conservation of the sequence and if a mutation is present in the conserved region there are high chances that it influences disease association.

As the dataset was very high dimensional so we applied dimensionality reduction algorithm namely Principal Component Analysis for this study. It reduces high dimensional data to low dimensional data by preserving the variance. It begins by first standardizing the data that normalizes the data using normal distribution. It creates a covariance matrix that computes the correlation between the features later the PCA components were generated that were ranked using eigen values. Each component captures the variance in the data.

### One class classification models

This study will employ one class classification to determine the disease associated peptides. One class SVM (OCSVM) is a semi-supervised algorithm, that tries to identify a single class and rejects the other. OCSVM works by learning a decision boundary that tries to differentiate the inliers with the outliers. We experimented with both ‘linear’ and ‘rbf’ (radial base function) kernels. Linear kernel assumes that the data is linearly separable while ‘rbf’ captures the non-linear relationship in the data.

Second we applied Isolation Forest to determine the outliers and inliers of the positively labelled data. This is also an unsupervised learning algorithm that is also used for anomaly detection. It randomly selected the features and split values, construct trees that measures how fast the samples are isolated. Those data points which were easily isolated are referred as outliers. As the input contains only positive labelled data we have identified the inliers that represents the majority of the data with a label of 1 and label the outliers as -1 that represents the anomalies.

Lastly, we trained autoencoders an unsupervised neural network to further model the positively labelled inliers. Autoencoders first uses the inbuilt encoders that compresses the input data into low dimensional data which was later decoded by the decoders that reconstructs the original data from the compressed data. Autoencoders uses reconstruction error as the performance measure by minimizing it, the autoencoders learns the significant features. To avoid overfitting we trained our autoencoders with epochs to 50. The trained model will then be used to assess the new data points .

### Informative feature selection

After applying one class classification models we have applied SHAP which is the model-agnostic interpretation as a feature selection mechanism. This method is also called as model addictive explanation approach that gives importance of each feature based on its contribution. SHAP or Shapley approximate the solution by exploring and using local explainability to build surrogate models for black-box machine learning models. In order to determine the importance of the feature, SHAP tries to change the input and tests model’s prediction if the prediction changes that means this feature is important if it does not that means that the feature may not be an important predictor^18^.

### Disease peptide anomaly score (DPAS) framework

To prioritize candidate peptides selection associated with disease we have introduces a scoring scheme named as Disease Peptide Anomaly Score (DPAS). This metric combines both model driven reconstruction errors and feature based importance values to quantify each peptide.

Formally, the DPAS is defined as

$$\:DPAS=\alpha\:\:\times\:\:Normalized\:Reconstrcution\:error+\beta\:\times\:Feature\:Importance$$

Where α and β are user defined weights that controls the contribution of of reconstruction errors and features. These scores helps us to prioritize the top ranked disease associated peptides.

### Functional annotation of prioritized peptides

To examine the biological relevance of the predicted disease associated peptides we deployed a downstream analysis that involves functional annotation of the peptides. First, we applied our Disease Peptide Anomaly Score (DPAS) to rank peptides by their likelihood of disease association. The top selected peptides, based on DPAS scores, were chosen for further analysis. Each peptide sequence was scanned in ScanProsite tool^19^to annotate the peptides. The tool searched for matches against known PROSITE patterns and profiles, including functionally annotated short linear motifs (SLiMs), post-translational modification (PTM) signatures, metal-binding motifs, and enzyme active site patterns.

## Results and discussion

In this study we have employed one-class classification approach to predict disease associated peptides that addresses the challenge of generating unreliable negative dataset. The positive labelled dataset was generated from XMAN database, a comprehensive repository of mutated peptides. Using this database, we generated a set of mutated peptide sequences making it appropriate for studying disease associated peptides. The diverse set of features were extracted that combines various sequence based features together. For classification, we employed One-Class SVM (OCSVM), Isolation Forest, and Autoencoders, and the performance was evaluated using mean reconstruction error for autoencoders while decision function for One-Class SVM, we, which provides the signed distance of each sample from the learned boundary. Similarly, for Isolation Forest, we use the model’s native score_samples, which reflects the anomaly score based on isolation depth as performance metrics. Mean reconstruction error provides a quantitative measure based on the separation of inliers meaning disease associated peptides with outliers meaning potential non-disease peptides. Inlier peptides should have a lower reconstruction errors, while outlier peptides, which deviates from expected characteristics, show significantly higher errors. This approach successfully apprehends the sequence variations that are unique to disease-associated peptides that also helps in biological significance.

As the data was high dimensional we have applied PCA that reduces the dimensions of the dataset.

**Fig. 2:**
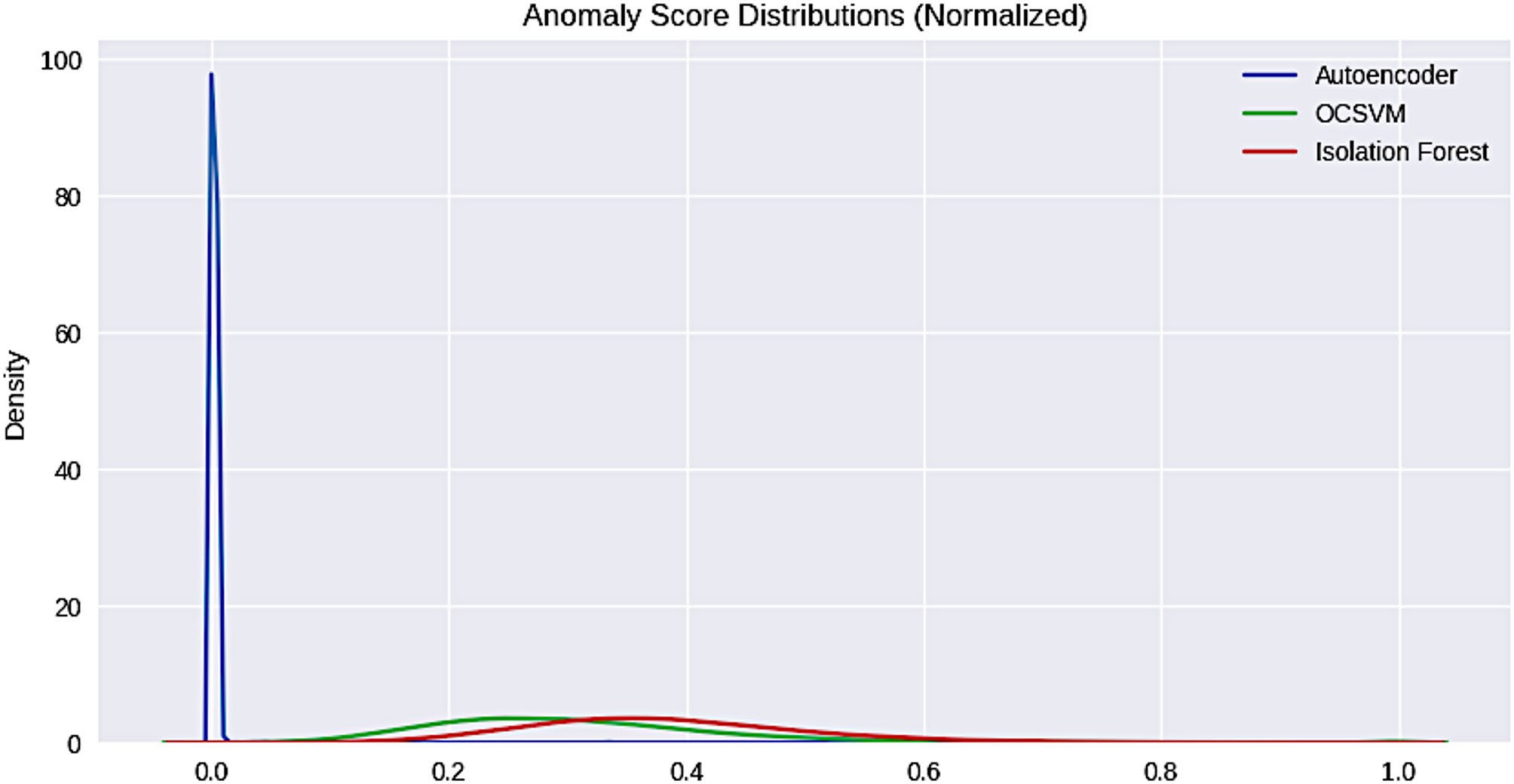
Anomaly Scores Distribution of the three methods.

The contribution of the input features in PCs are provided in the supplementary table S1 that explains the variance and important features included for classification. As stated earlier, for evaluating the classifiers we have used mean reconstruction error for both inliers and outliers. This is calculated by comparing the original data to the PCA-reconstructed data. The reconstruction error computes the difference between the input and reconstructed output representing how good the model apprehends the structure of the data. The Fig. 2 plots the cluster data for OCSVM, Isolation Forest, and Autoencoders. The features selected by each PCA highlighted the contribution of features with variance measures. PC1 indicates that PCP_PC (0.232407), Volume_Measure (0.229298), and Hydrophobicity_Measure (0.217602) were the top contributors to PC1, capturing the primary variance in the dataset. Meanwhile, PCP_NT (0.282786) and PCP_NC (0.248415) contributed significantly to PC2. PCP_AL negatively influenced PC1, indicating an inverse relationship with the dominant trend. Meanwhile the mutated sequences were used to build this model, the sequence-based properties are fundamental for understanding the role of mutations in disease association. Differences in volume measure, hydrophobicity, and amino acid composition between the mutated and normal sequences may significantly contribute to disease development.

To ensure consistent comparison among the three learning methods, we compared them using normalized anomaly scores computed for all samples. The Autoencoder achieved a mean normalized score of 0.00306 and a median of 0.00203, with a 95th percentile threshold of 0.00432, indicating that the majority of inlier samples are tightly clustered with low anomaly scores. In comparison, OCSVM and Isolation Forest showed higher mean and median scores (0.3069 and 0.2894 for OCSVM, 0.3823 and 0.3714 for Isolation Forest), reflecting a broader distribution of anomaly scores. All three methods flagged roughly 5% of the samples as outliers based on the 95th percentile threshold, but the Autoencoder’s lower baseline scores and tighter inlier distribution suggest better discrimination between inliers and outliers. Overall, these results indicate that the Autoencoder provides more precise and interpretable anomaly scoring, making it well-suited for detecting disease-associated peptides in this dataset. The results are tabulated in Table 1 and Figured in Fig. 2.

**Table 1:** Comparison of anomaly detection methods on peptide features.

Model	Mean Score	Median Score	95th Percentile Threshold	Outliers(%) Top 5%
Autoencoders	0.00306	0.00203	0.00432	5.00%
OCSVM	0.30690	0.28944	0.52834	5.00%
Isolation Forest	0.38233	0.37141	0.59392	5.00%

As the classification results showed that autoencoders outperformed OCSVM and Isolation Forest in predicting disease-associated peptides, we have computed the mean reconstruction errors for inliers and outliers indicating a larger gap between them. The Autoencoders showed a higher mean reconstruction error for outliers as compared to inliers suggests that inliers are being well reconstructed and Autoencoder models are well trained as they are close to the learned structure of the data. This is because the inliers represents majority of the class closely aligns with the learned patterns of the data resulting in low mean reconstruction errors. Outliers are likely points that deviates from the main data distribution, leading to higher reconstruction errors. This is expected because the models are unable to accurately reconstruct these unusual data points.

**Fig. 3:**
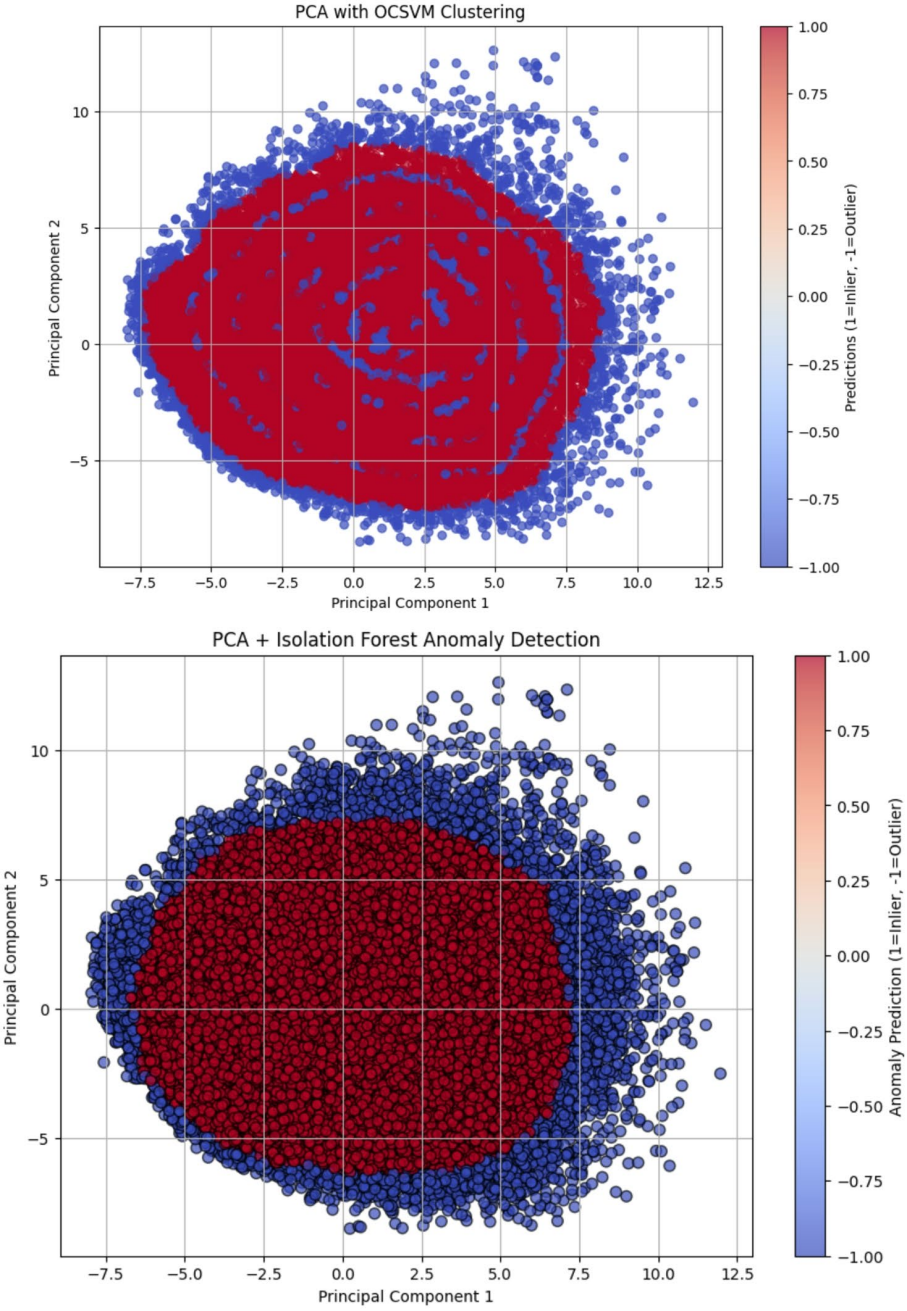
The distribution of Principal components and application of OCSVM, and Isolation Forest.

**Fig. 4:**
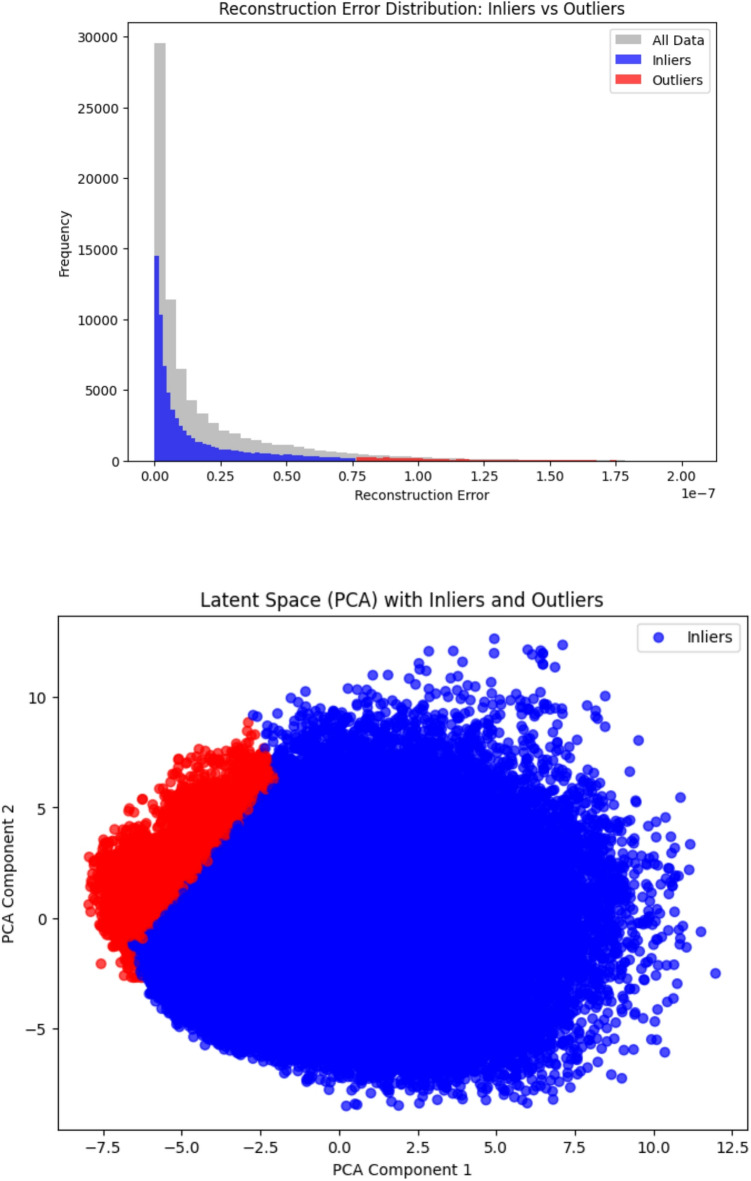
The Reconstruction Error distribution with threshold setting and PCA components via Autoencoders.

Furthermore, Autoencoders were further refined by adding a threshold based criteria to further enhance the separation of inliers with outliers. The value was chosen based on the distribution of reconstruction errors, corresponding approximately to the 95th percentile, we set a threshold value of 1.36 based on the reconstruction error. This threshold serves as a decision boundary with outliers above the boundary line and inliers below them. This threshold helps in enhancing the performance of the autoencoders. The results are tabulated in Table 2 and figured in Figs. 3 and 4.

Moreover, we have applied SHAP to identify the most informative features which can later be added in score calculation. SHAP identifies the top 3 informative features which are motifs and few physicochemical properties as the most contributing features. We have combined these with our autoencoder reconstruction errors to generate DPAS that prioritizes the top disease associated peptides. DPAS is the biologically informed score that not only adds anomalies for predicting disease associated peptides but justifies the prediction by adding both statistical and biological meaning to it. It adds the normalized anomalies reconstruction error that captures the anomalies behavior of the peptides with mutated peptides as input that naturally exhibits higher reconstruction errors thereby making them more deviated towards disease associations. Furthermore, DPAS also combines the important features identified by SHAP that provides a biologically informed weighting to the scoring scheme that highlights diseased peptides.

Lastly, the top 15 peptides ranked by DPAS were selected for downstream analysis. Each of these peptide sequences was queried against the ScanProsite tool for the functional annotation of the peptides. The results are summarized in Table 3 which suggest that most of the peptides are associated with functional motifs. The analysis highlighted the presence of post-translational modification motifs (e.g., phosphorylation), metal-binding motifs (e.g., CxxC), and nucleotide-binding motifs across several peptides. These findings suggest that the peptides may play roles in signal transduction, protein–protein interaction, or enzyme regulation. The presence of known motifs associated with enzymatic activity or regulatory roles strengthens their potential functional relevance in disease association or therapeutic targeting.

**Table 2:** Performance evaluation on threshold based trained Autoencoders.

Autoencoders	
Mean Reconstruction error for Inliers	0.913
Mean Reconstruction errors for outliers	5.90
Threshold	1.367
Total no of Inliers	69,013
Total no of outliers	7457
Accuracy of threshold based autoencoders	0.952

**Table 3:** Functional motif matches identified in peptide sequences using ScanProsite.

Peptide sequence	Matched Motif(s)	PROSITE ID	Motif description/putative function
qRWmEDL	[ST]-P	PS00005	Phosphorylation site (Ser/Thr-Pro)
lihFqGW	C-x(2)-C	PS00028	Zinc finger, C2H2-type
LENrCLa	G-x-G-x-x-G	PS00010	Nucleotide-binding motif (P-loop)
LSYGaGF	[LIVMFYW]-G-[DE]	PS00011	EF-hand calcium-binding region
iCGRkF	C-x(2)-[GAP]	PS00027	Possible disulfide bond-forming motif
RRiYDil	R-R-x(2)-[ST]	PS00008	cAMP- and cGMP-dependent protein kinase phosphorylation site
RRKmRqR	R-R-K	Custom Pattern	Putative nuclear localization signal (NLS-like)
pCGHtfC	C-x(3)-C	PS00029	Iron-sulfur binding domain
ihPvGWc	[ST]-x(2)-[DE]	PS00006	Casein kinase II phosphorylation site
HCqqkvW	H-x(3)-C	PS00031	Zinc finger-like pattern
QRTiAR	R-x-R	PS00020	Signal peptidase cleavage site
rYyEsWt	Y-x-E	Custom Pattern	Potential tyrosine phosphorylation region
CpyCGt	C-x-C-G	PS00030	Iron–sulfur cluster binding site
qCGyCtp	C-G-x-C	PS00025	CxxC metal binding motif
LLfShLS	S-x-[RK]	PS00007	Protein kinase C phosphorylation site

### Comparison with the existing methods

Previously reported studies focused on building supervised learning models that takes both positive and negative dataset for training. All these methods utilized mostly sequence based features to train models like SVM or in some cases deep learning embeddings with a well-defined negative dataset. This raises a challenge of generating reliable experimental non-disease peptides dataset. One of the studies DeepBP^20^employed ensemble deep learning model that achieved an accuracy of 92.6% for ACE inhibitor peptides and 77.9% for anticancer peptides. Similarly, pLMFPPred^21^utilized an imbalanced dataset and applied advance embedding techniques ESM2 which is a pre-trained language models- with SMOTE techniques and achieves an accuracy of 97.4%. Furthermore, studies like Multi-Peptide used transformer-based language models and Graph Neural Networks (GNNs) for hemolysis prediction, and reports an accuracy of 86.185%^22^. The comparison table is tabulated in Table 4.

While several existing studies have applied binary classification frameworks as mentioned earlier, all these models more or less depends on the availability of well-defined negative datasets. However, for disease-associated peptide prediction, biologically validated negative peptides do not exist, and negative examples used in previous work are typically heterogeneous, indirectly inferred, or constructed from unrelated peptide categories. Such things creates biasness in the model prediction and can also limit the generalizability of the model.

### Synthetic negative sample generation for evaluating one-class vs. supervised models

To further validate the performance of our one class classification models we have employed binary classification by generating a synthetic negative dataset and for that we adopted a column-wise random permutation strategy, which is widely used in anomaly detection and positive-only learning. For each feature column in the positive test feature vector (e.g., amino acid composition, dipeptide composition, or physicochemical descriptors), the values were randomly shuffled across samples while preserving the overall feature distribution. This process preserves the marginal distributions of individual features but also intentionally disrupts the biological and statistical dependencies between features. This synthetic negative dataset thus serves as a robust and reproducible reference for comparing and evaluating both traditional binary classifiers and one-class models. We evaluated two approaches for binary classification namely SVM and supervised autoencoders using both positive samples and synthetic negatives. The SVM achieved moderate performance, with high recall for positive samples but low precision for negatives, reflecting challenges in separating synthetic negatives from true positives in high-dimensional feature space. The autoencoders on the other hand, when trained as a binary classifier on the same dataset, performed comparably better, as it could leverage reconstruction-based feature learning to capture subtle differences between positives and synthetic negatives.

However, both methods showed limitations due to the artificial nature of the negative samples. These results suggest that while traditional binary classifiers like SVM provide a baseline, autoencoder-based approaches can offer improved discrimination in scenarios with synthetic or imperfect negative samples, supporting their use as complementary models for positive-only learning evaluation. The results are tabulated in Table 5.

**Table 4:** Performance comparison with the previous studies.

Study	Methodology	Features used	Performance measure	References
DeepBP: Ensemble Deep Learning Strategy for Bioactive Peptide Prediction	Ensemble deep learning models (CapsuleGAN, GRU, CNN)	Features extracted using the ESM-2 protein language model	ACE inhibitory peptides: 92.6% balanced accuracy; Anticancer peptides (ACPs): 77.9% accuracy	^20^
Predicting Antidiabetic Peptide Activity: A Machine Learning Approach	Logistic Regression, Support Vector Machines (SVM), Adaptive Boosting (AdaBoost)	Physicochemical properties, amino acid composition, and sequence-derived features	Not provided	^21^
Optimized Feature Learning for Anti-Inflammatory Peptide Prediction	Deep Neural Networks (DNN), Random Forest (RF)	Sequence-based features, physicochemical properties, and structural descriptors	DNN: 83.4% accuracy; RF: [Accuracy not specified]	^22^
Improved Prediction of Anti-Angiogenic Peptides Based on Machine Learning	Machine learning models with heuristic algorithms	4335 numeric features derived from 58 different feature types	Not provided	^23^
pLMFPPred: A Novel Approach for Accurate Prediction of Functional Peptides	Embeddings from pre-trained protein language model (ESM-2) with imbalanced learning techniques	ESM-2 embeddings, SMOTE-TOMEK data synthesis, Shapley value-based feature selection	Accuracy: 97.4%	^24^
Multi-Peptide: Multimodality Leveraged Language-Graph Learning of Peptide Properties	Combination of transformer-based language models and Graph Neural Networks (GNNs)	Sequence-based features, structural features, embeddings from PeptideBERT and GNN encoder	Hemolysis prediction: 86.185% accuracy	^25^

**Table 5:** Performance comparison with the previous studies.

Model	Accuracy (Pos/Neg)	Precision (Pos/Neg)	Recall (Pos/Neg)	F1-Score (Pos/Neg)
Autoencoders	0.60	0.57/0.76	0.90/0.31	0.69/0.44
SVM	0.50	0.23/0.78	0.23/0.67	0.35/0.77

## Conclusion

In this study we have proposed a one class classification approach to identify the disease associated peptides. The major challenge in classifying the disease associated and non-associated peptides is the identification of the negative dataset. Conventional methods usually uses the manually curated negative dataset that might create biased predications. Our study addresses this challenge by applying one class classification using only the positive labelled dataset. We applied three one class classification models namely SVM, Isolation Forest and Autoencoders. All these models are trained on positive labelled data and the performance was evaluated by computing the models capability of distinguishing the inliers with the outliers.

To enhance the biological interpretability we have introduced DPAS scoring metric that combines the reconstruction errors with SHAP identified most informative features allowing further prioritization of candidate peptides. Additionally the functional annotation analysis revealed the presence of known functional signatures such as phosphorylation sites, metal-binding domains, and other conserved motifs within several of the top-ranked peptides. These findings provide strong biological evidence for the potential involvement of these peptides in disease-related processes. The framework not only offers a more precise method for biomarker discovery and therapeutic development but also has the potential to be generalized to other biological domains, such as genomics, protein biology, and metabolomics, making it a valuable tool for advancing disease understanding and precision medicine. One key limitation of the current study is the lack of experimentally validated negative examples, which bounds the direct assessment of false positives. Furthermore, while the DPAS score enhances interpretability, it relies on assumptions from SHAP and reconstruction errors that may not fully capture complex biological nuances.

## Supplementary Information

Below is the link to the electronic supplementary material.

Supplementary Material 1

## Data Availability

The datasets used and/or analyzed during the current study available from the corresponding author on reasonable request.

## References

[1] Sperry, J. B. et al. Thermal stability assessment of peptide coupling reagents commonly used in pharmaceutical manufacturing. *Org. Process Res. Dev.* **22** (9), 1262–1275 (2018).

[2] Al-Azzam, S. et al. Peptides to combat viral infectious diseases. *Peptides* **134**, 170402 (2020). 32889022 10.1016/j.peptides.2020.170402 PMC7462603

[3] McGregor, D. P. Discovering and improving novel peptide therapeutics. *Curr. Opin. Pharmacol.* **8** (5), 616–619 (2008). 18602024 10.1016/j.coph.2008.06.002

[4] Li, F. et al. Positive-unlabelled learning of glycosylation sites in the human proteome. *BMC Bioinform.* **20**, 1–7 (2019). 10.1186/s12859-019-2700-1 PMC6404354 30841845

[5] Kılıç, C. & Tan, M. Positive unlabeled learning for deriving protein interaction networks. *Netw. Model. Anal. Health Inf. Bioinf.* **1**, 87–102 (2012).

[6] Gaffar, S., Hassan, M. T., Tayara, H. & Chong, K. T. IF-AIP: a machine learning method for the identification of anti-inflammatory peptides using multi-feature fusion strategy. *Comput. Biol. Med.* **168**, 107724 (2024). 37989075 10.1016/j.compbiomed.2023.107724

[7] Tyagi, A. et al. In Silico models for designing and discovering novel anticancer peptides. *Sci. Rep.* **3** (1), 2984 (2013). 24136089 10.1038/srep02984 PMC6505669

[8] Hajisharifi, Z., Piryaiee, M., Beigi, M. M., Behbahani, M. & Mohabatkar, H. Predicting anticancer peptides with Chou′ s Pseudo amino acid composition and investigating their mutagenicity via Ames test. *J. Theor. Biol.* **341**, 34–40 (2014). 24035842 10.1016/j.jtbi.2013.08.037

[9] Chen, W., Ding, H., Feng, P., Lin, H. & Chou, K. C. iACP: a sequence-based tool for identifying anticancer peptides. *Oncotarget* **7** (13), 16895 (2016). 26942877 10.18632/oncotarget.7815 PMC4941358

[10] XMAn A homo sapiens Mutated-Peptide database for the MS analysis of cancerous cell States. Xu Yang and Iulia M. *Lazar J. Proteome Res. 2014* **13** (12), 5486–5495 . 10.1021/pr5004467 25211293

[11] Manavalan, B., Shin, T. H., Kim, M. O., Lee, G. & AIPpred Sequence-Based prediction of Anti-inflammatory peptides using random forest. *Front. Pharmacol.* **9**, 276. 10.3389/fphar.2018.00276 (2018). PMID: 29636690; PMCID: PMC5881105. 29636690 10.3389/fphar.2018.00276 PMC5881105

[12] Yavuz, A. S., Ozer, B. & Sezerman, O. U. Pattern recognition for subfamily level classification of GPCRs using motif distillation and distinguishing power evaluation. *Pattern Recognitions Bioinf.* **vol, 7632**, 267–276 (2012).

[13] Kharakoz, D. P. Partial volumes and compressibilities of extended polypeptide chains in aqueous solution: additivity scheme and implication of protein unfolding at normal and high pressure, biochemistry, **36**, pp. 10276–10285, (1997). 10.1021/bi961781c 9254626

[14] Hopp, T. P. & Woods, K. R. Prediction of protein antigenic determinants from amino acid sequences, Proc Natl Acad Sci U S A, vol no. 78, pp. 3824–8, 1981. (1981). 10.1073/pnas.78.6.3824 PMC319665 6167991

[15] Pande et al. Pfeature: A tool for computing wide range of protein features and Building prediction models. *J. Comput. Biol. 2022 Oct.* **13**10.1089/cmb.2022.0241 (2022). 10.1089/cmb.2022.0241 36251780

[16] Timothy, L., Bailey, J., Johnson, C. E., Grant, W. S. & Noble The MEME suite. *Nucleic Acids Res.* **43** (W1), W39–W49 (2015). 25953851 10.1093/nar/gkv416 PMC4489269

[17] Bailey, T. L. & Gribskov, M. Combining evidence using p-values: application to sequence homology searches. *Bioinf. (Oxford England)*. **14** (1), 48–54 (1998). 10.1093/bioinformatics/14.1.48 9520501

[18] Marcílio, W. E. & Eler, D. M. From explanations to feature selection: assessing SHAP values as feature selection mechanism. In2020 33rd SIBGRAPI conference on Graphics, Patterns and Images (SIBGRAPI) 2020 Nov 7 (pp. 340–347). Ieee.

[19] De Castro, E. et al. ScanProsite: detection of PROSITE signature matches and ProRule-associated functional and structural residues in proteins. *Nucleic Acids Res.* **34** (suppl_2), W362–W365 (2006). 16845026 10.1093/nar/gkl124 PMC1538847

[20] Zhang, M., Zhou, J., Wang, X., Wang, X. & Ge, F. DeepBP: ensemble deep learning strategy for bioactive peptide prediction. *BMC Bioinform.* **25** (1), 352 (2024). 10.1186/s12859-024-05974-5 PMC11556071 39528950

[21] Cai, K. et al. Predicting antidiabetic peptide activity: A machine learning perspective on type 1 and type 2 diabetes. *Int. J. Mol. Sci.* **25** (18), 10020 (2024). 39337508 10.3390/ijms251810020 PMC11432216

[22] Khan, S. et al. Optimized feature learning for anti-inflammatory peptide prediction using parallel distributed computing. *Appl. Sci.* **13** (12), 7059 (2023).

[23] Lee, Y. C., Yu, J. C., Ni, K., Lin, Y. C. & Chen, C. T. Improved prediction of anti-angiogenic peptides based on machine learning models and comprehensive features from peptide sequences. *Sci. Rep.* **14** (1), 14387 (2024). 38909149 10.1038/s41598-024-65062-9 PMC11193773

[24] Ma, Z. et al. pLMFPPred: a novel approach for accurate prediction of functional peptides integrating embedding from pre-trained protein Language model and imbalanced learning. ArXiv Preprint ArXiv:2309.14404. Sep 25. (2023).

[25] Badrinarayanan, S., Guntuboina, C., Mollaei, P. & Barati Farimani, A. Multi-peptide: multimodality leveraged language-graph learning of peptide properties. *J. Chem. Inf. Model.* **65** (1), 83–91 (2024). 39700492 10.1021/acs.jcim.4c01443 PMC11733943

