# Supplementary material for: DPAS: disease-associated peptide anomaly score for identifying pathogenic peptides via one-class learning
Source: Sci Rep. 2026 Feb 15;16:9170. doi: 10.1038/s41598-026-40099-0 (PMC12996630; doi:10.1038/s41598-026-40099-0)
Supplement: Supplementary file 1 — Supplementary Material 1 [file 41598_2026_40099_MOESM1_ESM.docx]

| **Supplementary Table S1: Important Features and Their Contributions:** |
| --- |

PC1 PC2 Max_Contribution

PCP_NT -0.013571 0.282786 0.282786

PCP_NC 0.135628 0.248415 0.248415

PCP_PC 0.232407 -0.118691 0.232407

Volume_Measure 0.229298 -0.135070 0.229298

freqSeq[DE] 0.124337 0.220514 0.220514

Hydrophobicity_Measure 0.217602 0.184550 0.217602

PCP_AL -0.214963 -0.039565 0.214963

freqSeq[RKH] 0.196523 -0.099280 0.196523

E[AAC] 0.120232 0.185836 0.185836

SER_E 0.120384 0.183205 0.183205

RRI_E 0.118155 0.181994 0.181994

E 0.117040 0.177786 0.177786

PCP_HL 0.177054 -0.125696 0.177054

K[AAC] 0.163549 -0.035936 0.163549

SER_K 0.163183 -0.035114 0.163183

RRI_K 0.160508 -0.035637 0.160508

D[AAC] 0.065110 0.160378 0.160378

SER_D 0.065657 0.159069 0.159069

K 0.158751 -0.033990 0.158751

RRI_D 0.065018 0.157560 0.157560

D 0.064571 0.155449 0.155449

R[AAC] 0.140096 -0.071687 0.140096

SER_R 0.138991 -0.072145 0.138991

RRI_R 0.137506 -0.070984 0.137506

R 0.135159 -0.070504 0.135159

freqSeq[YF] -0.012072 -0.115051 0.115051

SER_G -0.110446 0.074571 0.110446

G[AAC] -0.110222 0.075287 0.110222

RRI_G -0.108571 0.072848 0.108571

G -0.107637 0.072369 0.107637

freqSeq[G] -0.107637 0.072369 0.107637

H[AAC] 0.070252 -0.100228 0.100228
